# Prussian Blue Nanoparticles Promoting Diabetic Bone Regeneration via Mitochondrial Recovery

**DOI:** 10.34133/bmef.0204

**Published:** 2025-12-22

**Authors:** Anqi Gu, An Lao, Weiqi Li, Ziyang Liu, Chuang Zhou, Jianqiang Cai, Qiang Chen, Kaili Lin, Lijuan Song, Xiangbing Wu, Jiaqiang Liu

**Affiliations:** ^1^ Department of Oral and Cranio-maxillofacial Surgery, Shanghai Ninth People’s Hospital, Shanghai Jiao Tong University School of Medicine; College of Stomatology, Shanghai Jiao Tong University; National Center for Stomatology; National Clinical Research Center for Oral Diseases; Shanghai Key Laboratory of Stomatology, Shanghai 200011, China.; ^2^ Department of Stomatology, Xin Hua Hospital, Shanghai Jiao Tong University School of Medicine, Shanghai 200092, China.; ^3^ Department of Implant Dentistry, Suzhou Stomatological Hospital, Suzhou 215005, China.; ^4^ Department of Periodontics, Suzhou Stomatological Hospital, Suzhou 215005, China.; ^5^ Department of Orthodontics, Suzhou Stomatological Hospital, Suzhou 215005, China.

## Abstract

**Objective:** This work aims to develop Prussian blue (PB) nanoparticles that mitigate bone marrow mesenchymal stem cell (BMSC) senescence and alleviate bone loss in type 2 diabetes (T2D). **Impact Statement:** PB nanozymes are established as a targeted therapeutic strategy for maintaining bone quality in T2D—addressing an unmet clinical need through innovative nanomaterial design. **Introduction:** Diabetes is associated with a higher risk of fractures through distinct mechanisms. Elevated blood sugar levels and excessive nutrition in T2D trigger reactive oxygen species (ROS) overproduction that impairs mitochondrial function, induces BMSC senescence, and compromises osteogenic potential. Engineered as artificial enzyme counterparts, nanozymes effectively eliminate ROS while circumventing the inherent constraints of natural antioxidant enzymes. **Methods:** PB nanoparticles were synthesized and fully characterized. BMSCs treated with high glucose plus palmitate–bovine serum albumin served as the diabetic cell model. The nanoparticles were evaluated for their capacity to scavenge ROS, modulate mitochondrial function, counteract cellular senescence, and restore osteogenic potential. Finally, their ability to attenuate bone loss was verified in a T2D mouse model. **Results:** We demonstrated that PB nanoparticles efficiently scavenge ROS, rebalance mitochondrial dynamics by up-regulating fusion proteins and down-regulating fission proteins, and restore membrane potential. These actions suppress BMSC senescence and revive osteogenic capacity, culminating in substantial attenuation of T2D-associated bone loss in vivo. **Conclusion:** These findings introduce a promising and innovative approach for managing bone quality in patients with T2D.

## Introduction

The global age-standardized prevalence of diabetes is expected to grow at an annualized rate of 3.31% between 2021 and 2050 [1]. Patients with diabetes have a significantly increased risk of fractures compared to normal individuals [2]. Skeletal fragility is a recognized, serious complication of type 2 diabetes (T2D) [3,4]. The senescence of bone-marrow-derived mesenchymal stem cells (MSCs) in the diabetic microenvironment critically contributes to the pathogenesis of compromised bone healing [5]. Despite the availability of numerous therapeutic agents for diabetes mellitus, their efficacy in promoting diabetic bone defect healing remains limited. Research indicates that the pathophysiology of diabetic bone loss involves highly complex mechanisms, encompassing mitochondrial dysfunction, excessive reactive oxygen species (ROS) production, cellular senescence, immune dysregulation, and disrupted bone deposition–resorption equilibrium [6,7].

Within the diabetic microenvironment, stem cells sense metabolic danger signals and exhibit functional impairment. These metabolic danger signals are recognized as triggers for the excessive production of ROS, alongside mitochondrial dysfunction [8]. Mitochondrial dysfunction is established as both a cause and a consequence of cellular senescence, critically contributing to feedback mechanisms that initiate and maintain the senescent phenotype. Subsequently, senescent cells generate increased ROS, exacerbating mitochondrial dysfunction and suppressing biogenesis, thereby reinforcing positive feedback loops [9,10]. Consequently, strategies targeting ROS removal and mitochondrial recovery to ameliorate aged microenvironments and senescent bone marrow mesenchymal stem cells (BMSCs) represent promising novel approaches for enhancing bone defect healing in T2D patients.

Enzymes catalyze diverse physiological metabolic processes, yet inherent limitations, such as high preparation and storage costs and sensitivity to external environmental factors, hamper the broad clinical application of natural enzymes [11]. In recent years, nanozyme applications in biomedicine have attracted marked attention [12]. Artificial nanozymes demonstrate distinct advantages over natural enzymes, including enhanced stability, superior durability, facile synthetic feasibility, and reduced production costs [11].

In addition to serving as a drug carrier, contrast agent, and photothermal converter, Food and Drug Administration-approved Prussian blue (PB) mimics peroxidase, catalase, and superoxide dismutase activities, offering therapeutic potential through the elimination of excess ROS and mitochondrial function regulation in the T2D microenvironment [13,14]. Previous studies have demonstrated that thermosensitive hydrogels or microneedle patches delivering PB nanozymes can accelerate diabetic wound healing by targeting mitochondrial stress and scavenging ROS [15,16]. However, the therapeutic potential of PB against diabetic-microenvironment-induced bone loss and compromised osteogenesis remains unexplored.

Herein, we aimed to utilize PB to develop a functional strategy for alleviating the senescence of BMSCs in the diabetic microenvironment. We investigated the effects of PB in maintaining mitochondrial function and reducing ROS leakage and accumulation. Finally, both in vitro and in vivo studies were used to evaluate the effects of this nanozyme in reducing diabetic bone loss.

## Results

### Characterizations of PB

**Fig. 1.**
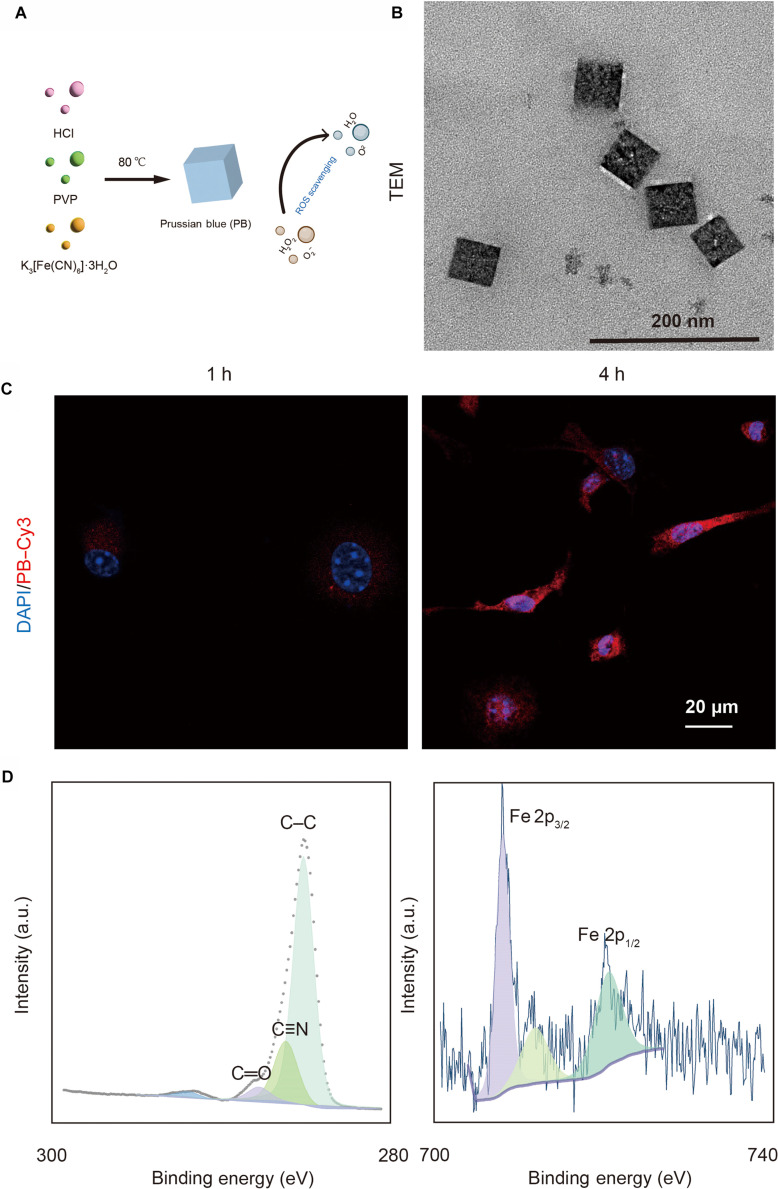
Characterizations of Prussian blue (PB) nanoparticles. (A) Schematic illustration of the preparation of PB. (B) Transmission electron microscopy (TEM) images of PB. (C) Confocal laser scanning microscope (CLSM) images of bone marrow mesenchymal stem cells (BMSCs) treated with PB for 1 and 4 h. (D) C 1s and Fe 2p x-ray photoelectron spectroscopy (XPS) spectra. PVP, polyvinylpyrrolidone; ROS, reactive oxygen species; DAPI, 4′,6-diamidino-2-phenylindole.

PB was synthesized according to a previously reported method (Fig. 1A) [17]. The morphology of PB was observed by transmission electron microscopy. The PB particles were cubic shaped with a diameter of ∼100 nm (Fig. 1B). To evaluate PB uptake by BMSCs, Cy3-labeled PB were co-cultured with cells for 1 and 4 h. The results demonstrated noticeably higher PB internalization after 4 h compared to that after 1 h (Fig. 1C). To deeply investigate the elemental composition and valence states on the surface of Prussian blue nanoparticles (PBNPs), x-ray photoelectron spectroscopy analysis was performed. As shown in Fig. 1D, the 3 components fitted in the C 1s spectrum at 284.8, 285.9, and 287.5 eV correspond to C–C, C≡N, and C=O bonds, respectively. The results also indicate that the Fe 2p peaks of PB can be deconvoluted into 3 component peaks. The peaks at binding energies of 708.7 and 721.8 eV belong to the characteristic Fe^2+^ 2p_3/2_ and Fe^2+^ 2p_1/2_ peaks, respectively, while the peak at 712.8 eV likely corresponds to Fe_2_O_3_, formed by the oxidation of Fe^2+^. The remaining peaks are satellite peaks of the material. Analysis of the x-ray photoelectron spectroscopy spectra confirms the presence of [Fe(CN)_6_]^3−^ units within the material, demonstrating the successful construction of PBNPs.

### PB regulates mitochondrial function

**Fig. 2.**
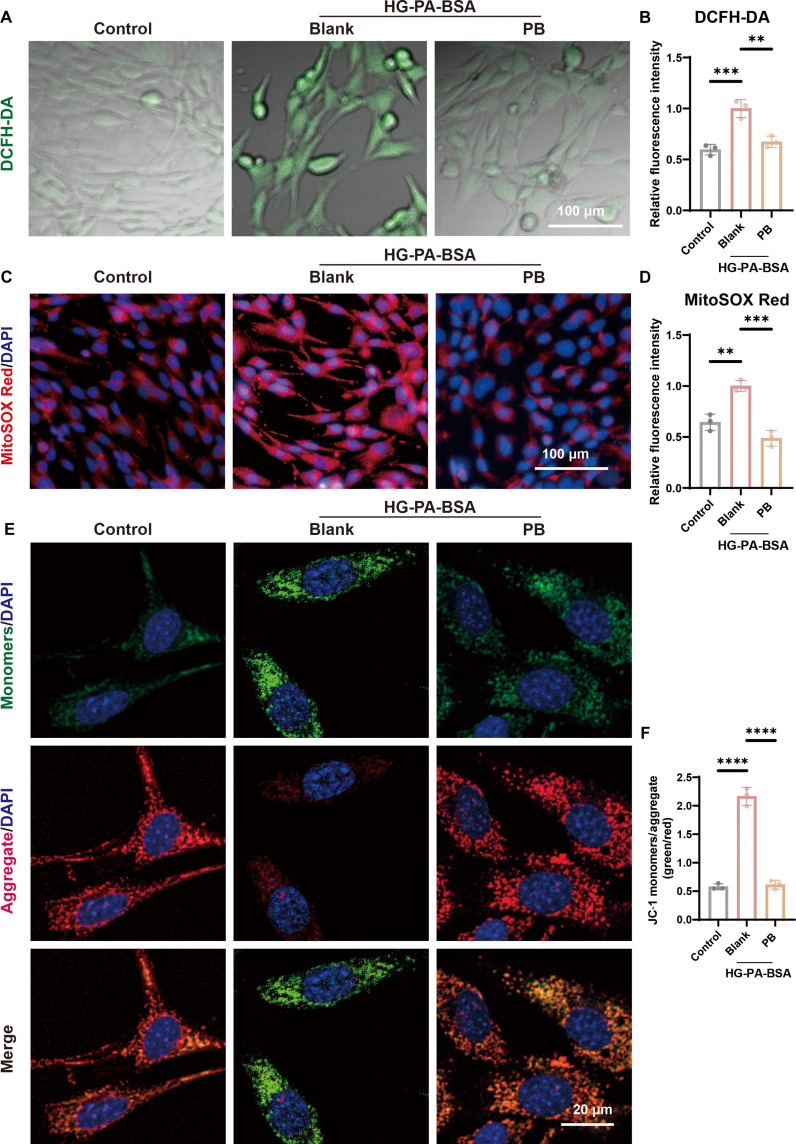
PB nanoparticles recover mitochondria. (A and B) Representative immunofluorescent images and quantitative analyses of 2′,7′-dichlorodihydrofluorescein diacetate (DCFH-DA) staining for intracellular ROS (*n* = 3). (C and D) Representative immunofluorescent images and quantitative analyses of MitoSOX Red (*n* = 3). (E and F) Representative immunofluorescent images and quantitative analyses of JC-1 (*n* = 3). **P < 0.01; ***P < 0.001; ****P < 0.0001. HG-PA-BSA, high glucose plus palmitate–bovine serum albumin.

Diabetes-associated cellular senescence involves mitochondrial dysfunction driven by excessive ROS production [18]. Intracellular ROS were quantified using the fluorogenic probe 2′,7′-dichlorodihydrofluorescein diacetate (DCFH-DA). Analysis revealed that high glucose plus palmitate–bovine serum albumin (HG-PA-BSA) treatment significantly elevated intracellular ROS levels compared to those under normal conditions, whereas nanoparticle administration significantly attenuated this increase (Fig. 2A and B). To specifically assess mitochondrial ROS, we employed the highly selective fluorescent dye MitoSOX Red (Fig. 2C and D). Concurrently, mitochondrial membrane potential (ΔΨm) was evaluated via JC-1 staining, demonstrating that PB treatment decreased the green/red fluorescence intensity ratio—indicative of ΔΨm recovery (Fig. 2E and F).

### PB regulates mitochondrial morphology

**Fig. 3.**
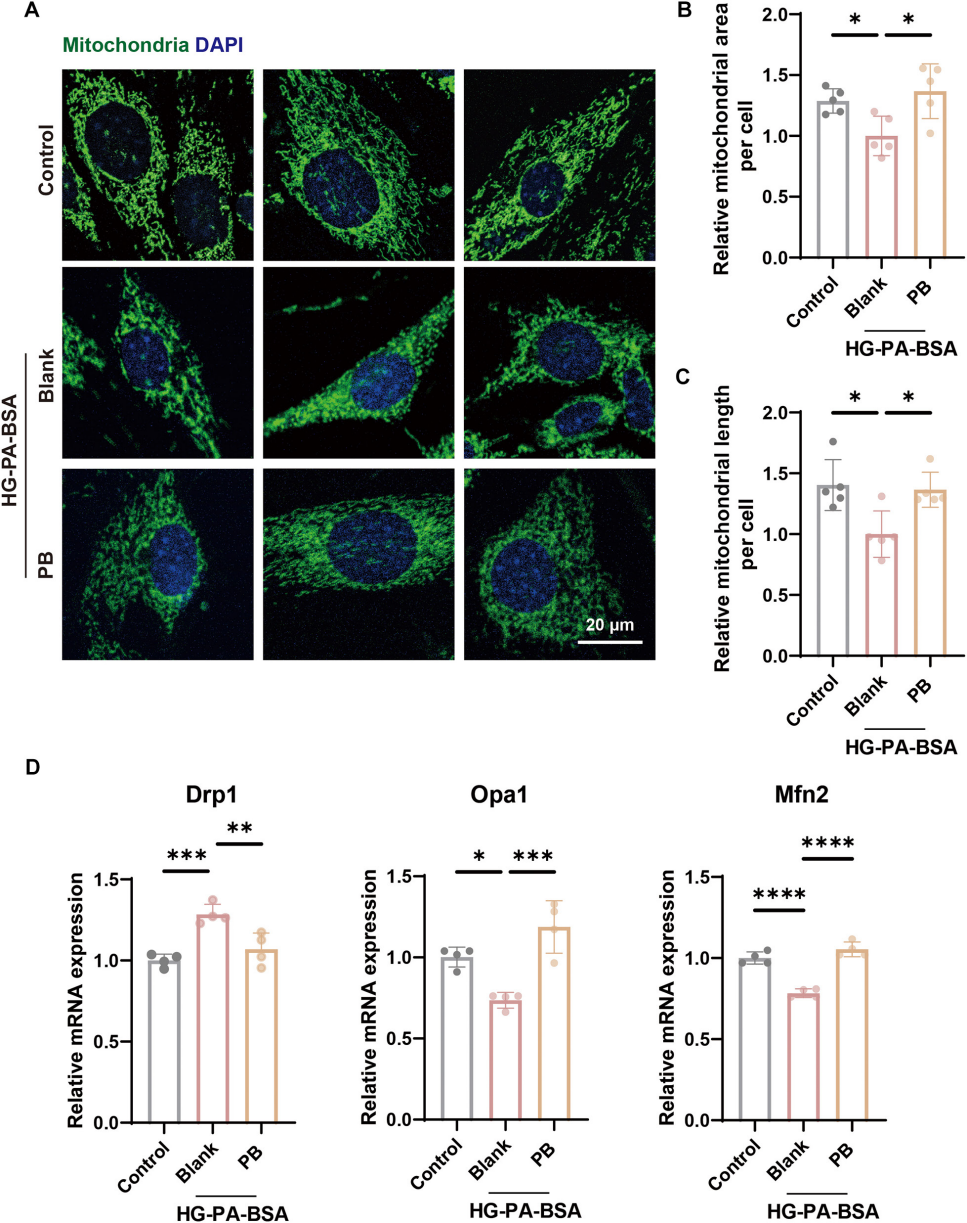
The changes in mitochondrial morphology. (A) Representative immunofluorescent images of MitoTracker Green. (B) Quantification of the mitochondrial content according to MitoTracker Green staining (*n* = 5). (C) Quantification of the mitochondrial length according to MitoTracker Green staining (*n* = 5). (D) Quantitative analyses of gene expression levels of Drp1 (dynamin-related protein 1), Opa1 (optic atrophy 1), and Mfn2 (mitofusin 2) of BMSCs (*n* = 4). *P < 0.05; **P < 0.01; ***P < 0.001; ****P < 0.0001. mRNA, messenger RNA.

Mitochondrial fission–fusion dynamics directly correlate with organellar activity, a relationship governed by the intracellular redox state modulated through metabolic processes that regulate ROS [19]. Perturbation of mitochondrial dynamics can lead to cellular senescence [20]. Staining of MitoTracker Green on BMSCs demonstrated that HG-PA-BSA treatment reduced mitochondrial area per cell and turned mitochondria into shorter morphologies, indicating excessive mitochondrial fission compared to that in the control group. PBNPs significantly regulated mitochondrial morphology with the increase in mitochondrial content and length of mitochondria, indicating PB promoting mitochondrial fusion (Fig. 3A to C). To further confirm mitochondrial dynamics activation, the expression of key fusion- and fission-related genes was assessed. PB-exposed BMSCs exhibited a significant up-regulation of the fusion genes Opa1 (optic atrophy 1) and Mfn2 (mitofusin 2) and a concurrent down-regulation of the fission gene Drp1 (dynamin-related protein 1) compared with the HG-PA-BSA-treated group (Fig. 3D).

### PB alleviated cellular senescence and restored osteogenic potential

**Fig. 4.**
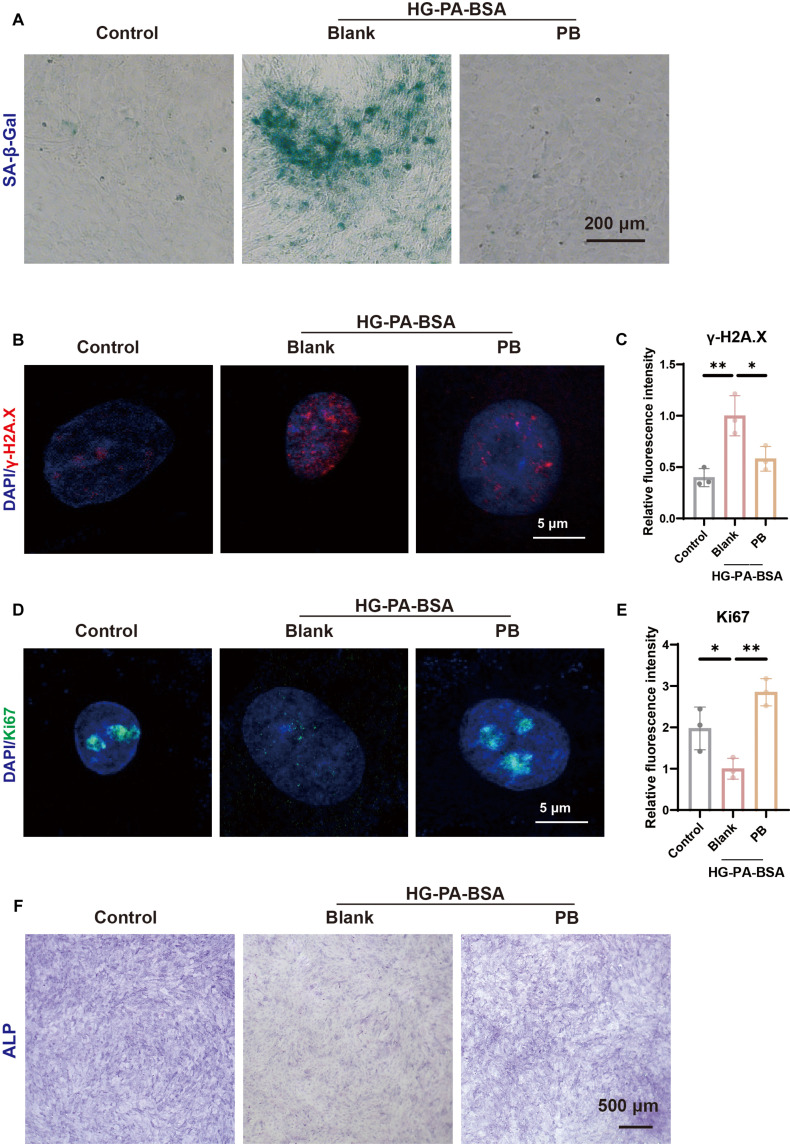
PB alleviated BMSC senescence and restored osteogenic potential. (A) Representative images of senescence-associated beta-galactosidase (SA-β-Gal) staining of BMSCs in different groups. (B and C) Representative fluorescent images and quantitative analyses of γ-H2A.X of BMSCs in different groups (*n* = 3). (D and E) Representative fluorescent images and quantitative analyses of Ki67 of BMSCs in different groups (*n* = 3). (F) Representative images of alkaline phosphatase (ALP) staining in BMSCs in different groups. *P < 0.05; **P < 0.01.

The diabetic microenvironment caused cellular senescence. Studies have reported that diabetes mellitus affects the senescence of MSCs, which is connected to the properties and functions of cells. Senescent cells were defined by elevated senescence-associated beta-galactosidase (SA-β-Gal) activity, DNA damage, cell cycle arrest, and senescence-associated secretory phenotype secretion [21]. HG-PA-BSA induced BMSCs’ senescence, as shown by SA-β-Gal staining, while PB treatment could strongly inhibit cellular senescence (Fig. 4A). The cell proliferation marker Ki67 and DNA damage marker γ-H2A.X also provided sufficient proof to support the claim that PB treatment could contain senescent BMSCs (Fig. 4B to E). The aging process impairs MSC function, leading to a marked decline in osteogenic capacity [22]. After a 7-d period of osteogenic induction, the alkaline phosphatase (ALP) staining reveals that PBNPs reversed the suppression of ALP activity induced by HG-PA-BSA treatment (Fig. 4F).

### PB ameliorated T2D-induced bone loss

**Fig. 5.**
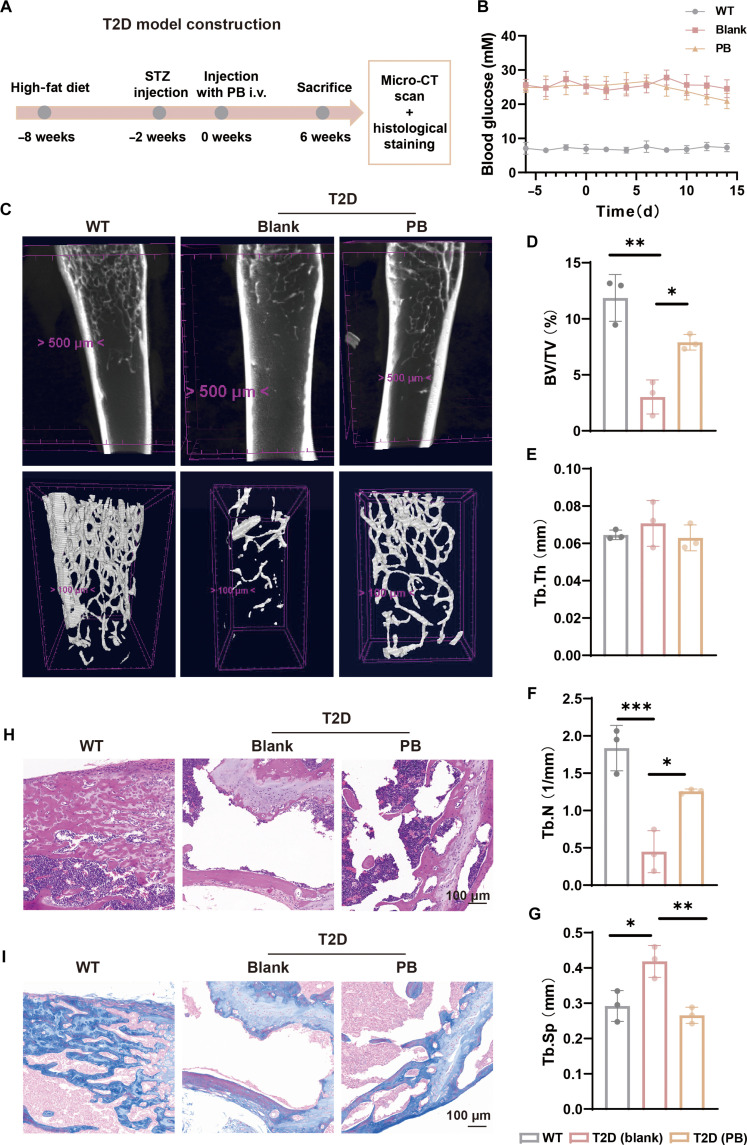
PB ameliorated type 2 diabetes (T2D)-induced bone loss. (A) Schematic representation of the T2D mouse model. (B) Blood glucose changes in mice over time (*n* = 3). (C) The microcomputed tomographic scans of trabecular bone mass. Quantification of bone-to-tissue volume ratio (BV/TV) (D), trabecular thickness (Tb.Th) (E), trabecular number (Tb.N) (F), and trabecular separation (Tb.Sp) (G) (*n* = 3). (H) Representative hematoxylin and eosin (H&E) staining of distal femur specimens. (I) Representative Masson staining of distal femur specimens. *P < 0.05; **P < 0.01; ***P < 0.001. STZ, streptozotocin; i.v., intravenous; CT, computed tomography; WT, without treatment.

We tested the therapeutic effect of the PBNPs by establishing a high-fat-diet (HFD)-induced T2D mouse model. The study involved intravenously injecting HFD-induced T2D mice with nanovesicles for a duration of 3 weeks to assess the treatment’s impact on T2D-related bone loss (Fig. 5A). Every-other-day blood glucose monitoring verified adequate establishment of the T2D model (Fig. 5B). Parameters including bone-to-tissue volume ratio, trabecular separation, connectivity density, trabecular number, and structure model index obtained using animal computed tomography indicate significant bone loss in the T2D group (Fig. 5C). PB treatment significantly attenuated bone loss, thickened trabeculae, and preserved trabecular architecture in T2D mice (Fig. 5D to G). Histological analysis via hematoxylin and eosin (Fig. 5H) and Masson’s trichrome staining (Fig. 5I) demonstrated that PB effectively mitigated T2D-induced bone loss. Histopathological analysis revealed that all treated animals exhibited normal tissue architecture, with no evidence of pathological lesions, confirming the clinical translational potential of PB (Fig. S1). Overall, PB normalized the microenvironment to alleviate T2D bone loss in vivo.

## Discussion

PBNPs have emerged as promising therapeutic agents in biomedicine, leveraging their enzyme-mimetic activities, antioxidant potency, and immunomodulatory functions. Evidence indicates that PBNPs can remodel pathological microenvironments to support tissue regeneration, as demonstrated by their efficacy in alveolar bone defects to combat hypoxia and promote osteogenesis [23], in tendinopathy to scavenge ROS and polarize macrophages toward a restorative phenotype [24], and in intestinal repair where PB-integrated hydrogels enhance healing via antioxidative and anti-inflammatory mechanisms [25]. Despite these advances, the role of PBNPs in the diabetic bone microenvironment, particularly regarding mitochondrial dynamics, cellular senescence, and osteogenic regeneration, has remained unclear.

This study provides the first demonstration that PBNPs alleviate BMSC senescence and promote bone regeneration under diabetic conditions through mitochondrial regulation. Our results show that PBNPs not only effectively scavenge ROS but also restore mitochondrial homeostasis by rebalancing fusion–fission dynamics through specific up-regulation of OPA1 and MFN2 and down-regulation of DRP1 while concurrently recovering mitochondrial membrane potential (ΔΨm). The resulting reversal of mitochondrial fragmentation consequently suppressed BMSC senescence, as indicated by reduced SA-β-Gal activity, elevated Ki67 expression, and diminished γ-H2A.X foci. This restoration of mitochondrial function further rejuvenated the osteogenic potential of BMSCs, marked by increased ALP activity. Consistent with these cellular effects, PBNP treatment significantly attenuated T2D-related bone loss in vivo.

The findings of this study align with recent advances in the mitochondrial-aging research axis. For instance, Wang et al. [10] demonstrated that CeO_2_ nanozymes attenuate T2D-related bone aging by improving mitochondrial function. Huang et al. [26] developed a physical thermal therapy platform that enhances aged bone healing through the reinforcement of mitochondrial membrane integrity. Building on these insights, we propose that PB acts as a pleiotropic nanozyme that not only scavenges ROS but also remodels the mitochondrial network via transcriptional regulation of dynamics-related genes. This dual mechanism disrupts the self-propagating “ROS–mitochondrial damage–cellular senescence” cycle, offering a novel therapeutic strategy for diabetic bone regeneration.

We further hypothesize that the nuclear factor erythroid 2-related factor 2/sirtuin 1 (NRF2/SIRT1) signaling axis may act as an upstream mechanism through which PBNPs exert antioxidant and mitochondrial-protective effects, thereby promoting osteogenesis. Previous studies have demonstrated that NRF2 sustains MSC self-renewal and osteogenic potential by up-regulating SIRT1, and decline in age-related SIRT1 activity disrupts mitochondrial dynamics and homeostasis [27,28]. The observed up-regulation of the mitochondrial fusion factor OPA1 and MFN2, together with the down-regulation of the fission factor DRP1, supports the notion that the NRF2/SIRT1 axis represents a critical molecular conduit for these protective effects. Future studies employing transcriptomic and proteomic approaches should delineate the contributions of these pathways, providing a mechanistic foundation for optimizing PB-based nanotherapeutics in metabolic bone disorders.

## Conclusion

PB effectively mitigated bone loss in diabetic mice through mitochondrial-targeted therapy. It restored mitochondrial membrane potential and improved mitochondrial morphology in BMSCs while also suppressing stem cell senescence. In vivo experiments further demonstrated its capacity to promote bone regeneration in the diabetic mouse model.

## Materials and Methods

### Synthesis of PB

PB nanovesicles were synthesized using potassium ferricyanide (K_3_[Fe(CN)_6_], 226.7 mg), polyvinylpyrrolidone (3 mg), and hydrochloric acid (HCl, 35 μl), all sourced from Shanghai Aladdin Biochemical Technology. K_3_[Fe(CN)_6_] and polyvinylpyrrolidone were dissolved in ultrapure water (40 ml) under room-temperature stirring. Concentrated HCl was added to the mixture under vigorous agitation, followed by heating at 80 °C for 20 h. The products were isolated via ultracentrifugation, washed repeatedly with ultrapure water, and collected as PB after vacuum freeze-drying.

### In vitro osteogenic differentiation of BMSCs

The femur and tibia of 3-week-old Sprague–Dawley rats (Shanghai Sippr-BK Laboratory Animal Co. Ltd) were collected. BMSCs from the femur and tibia were cultured with alpha minimum essential medium containing 10% (v/v) fetal bovine serum and 1% penicillin–streptomycin in a 5% CO_2_ incubator at 37 °C. The medium was then transferred into the osteogenic induction medium, which was alpha minimum essential medium containing 10% (v/v) fetal bovine serum, 1% penicillin–streptomycin, 50 mg ml^−1^ l-ascorbic acid, 10 mM β-glycerophosphate, and 100 nM dexamethasone.

### Immunofluorescence staining and confocal microscopy

Immunofluorescence staining assessed typical protein expression. Samples were fixed in 4% paraformaldehyde (30 min), permeabilized with 0.5% Triton X-100 (15 min), and blocked in 1% BSA (60 min). Following primary antibody incubation at 4 °C for 12 h, samples were treated with secondary antibody (1 h) and counterstained with 4′,6-diamidino-2-phenylindole (10 min, room temperature). Imaging was performed using a Zeiss LSM880 microscope.

### ROS generation and scavenging potential evaluation

The following experiments were conducted according to the kit manufacturers’ instructions: DCFH-DA (Yeasen Biotech Co, Ltd, Shanghai, China) staining, JC-1 assays (Yeasen Biotech Co, Ltd, Shanghai, China), and MitoSOX Red assays (Thermo Fisher Scientific).

### Real-time quantitative PCR

Total RNA from BMSCs was extracted using TRIzol reagent. Then, complementary DNA was synthesized with Primer-Script RT reagent kit. Real-time quantitative polymerase chain reaction (PCR) was conducted using the TB Green Premix Ex Taq kit in LightCycler 96 Real-Time PCR System (Roche, Switzerland). Gene expression was normalized to β-actin.

### T2D diabetic mouse model

Six-week-old male mice were brought from Shanghai Sippr-BK Laboratory Animal Co. Ltd (China). Ethical approval was obtained from the Laboratory Animal Ethical Committee of Shanghai Ninth People’s Hospital, Shanghai Jiao Tong University School of Medicine (No. SH9H-2024-A1016-1) for all animal experiments. We used an HFD (60% cal/fat, D12492; Research Diets) to feed for 8 weeks and made mice receive intraperitoneal injection of streptozotocin (50 mg kg^−1^, Sigma) dissolved in citrate buffer (pH 4.5). Then, we selected the mice with blood glucose higher than 16.7 mmol l^−1^ and observed them for stable hyperglycemia for 1 week, indicating successful establishment of the T2D model.

### Histological analysis

The bone tissues were fixed in 4% paraformaldehyde. Then, tissue slices were stained with hematoxylin and eosin and Masson’s trichrome.

### Statistical analysis

All data analyses were performed by the GraphPad Prism 6.0 software. Statistical analysis was performed by one-way analysis of variance. Differences at *P* < 0.05 were considered significant.

## Data Availability

Data will be made available on request.
