# Supplementary figures and images for: Prussian Blue Nanoparticles Promoting Diabetic Bone Regeneration via Mitochondrial Recovery
Source: BME Front. 2025 Dec 22;6:0204. doi: 10.34133/bmef.0204 (PMC12719558; doi:10.34133/bmef.0204)

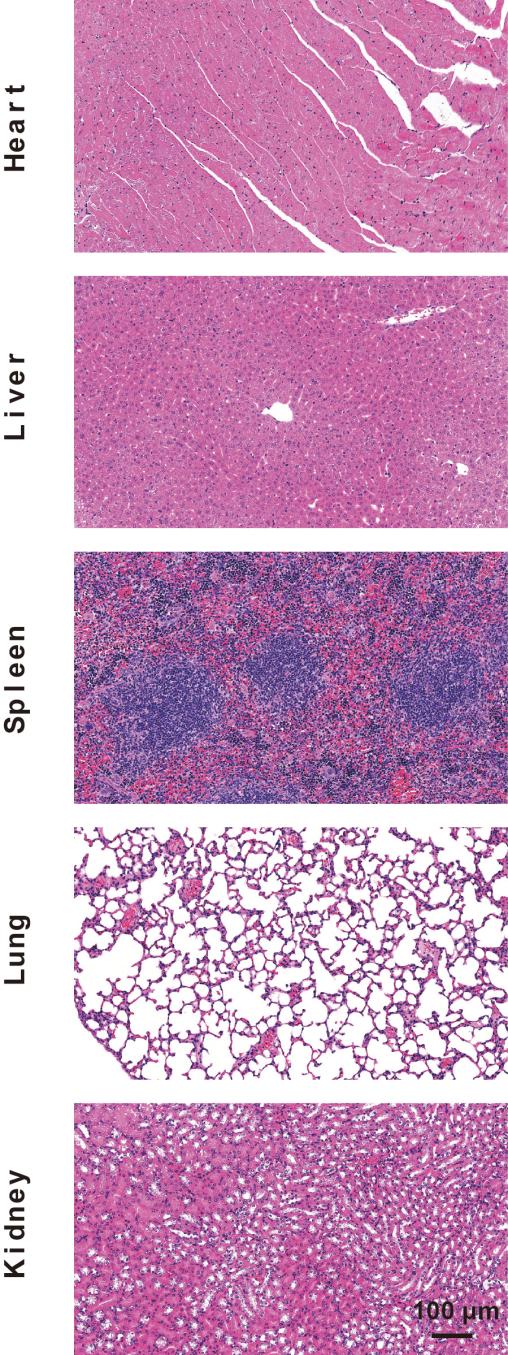


Figure.S1 Representative H&E staining images of major organs.

Supplement: Supplementary 1 — Fig. S1 [file bmef.0204.f1.docx]
